# Bifunction-Integrated Dielectric Nanolayers of Fluoropolymers with Electrowetting Effects

**DOI:** 10.3390/ma11122474

**Published:** 2018-12-05

**Authors:** Hao Wu, Hao Li, Ahmad Umar, Yao Wang, Guofu Zhou

**Affiliations:** 1 Guangdong Provincial Key Laboratory of Optical Information Materials and Technology & Institute of Electronic Paper Displays, National Center for International Research on Green Optoelectronics, South China Academy of Advanced Optoelectronics, South China Normal University, Guangzhou 510006, China; wuhao@scnu.edu.cn; 2 Physics of Complex Fluids, Faculty of Science and Technology, MESA+ Institute for Nanotechnology, University of Twente, 7500AE Enschede, The Netherlands; 3 Shenzhen Guohua Optoelectronics Tech. Co. Ltd., Shenzhen 518110, China; 4 Academy of Shenzhen Guohua Optoelectronics, Shenzhen 518110, China; 5 Department of Chemistry, Faculty of Science and Arts and Promising Centre for Sensors and Electronic Devices, Najran University, Najran 11001, Kingdom of Saudi Arabia; ahmadumar786@gmail.com

**Keywords:** electrowetting, fluoropolymers, dielectric layer, dielectric strength, contact angle

## Abstract

Fluoropolymers play an essential role in electrowetting (EW) systems. However, no fluoropolymer possesses the desirable properties of both hydrophobicity and dielectric strength. In this study, for the first time, we report the integration of two representative fluoropolymers—namely, Teflon AF (AF 1600X) and Cytop (Cytop 809A)—into one bifunctionalized dielectric nanolayer. Within this nanolayer, both the superior hydrophobicity of Teflon AF and the excellent dielectric strength of Cytop were able to be retained. Each composed of a 0.5 μm Cytop bottom layer and a 0.06 μm Teflon AF top layer, the fabricated composite nanolayers showed a high withstand voltage of ~70 V (a dielectric strength of 125 V/μm) and a high water contact angle of ~120°. The electrowetting and dielectric properties of various film thicknesses were also systemically investigated. Through detailed study, it was observed that the thicker Teflon AF top layers produced no obvious enhancement of the Cytop/Teflon AF stack.

## 1. Introduction

Electrowetting (EW) refers to the phenomenon of altering the surface wettability of an electrode or dielectric layer with an applied electric field [1,2]. As an approach to manipulating minute fluids, electrowetting has attracted a great deal of attention for its application within reflective display devices [3,4], lab-on-a-chip systems [5,6], and optic lenses [7,8]. The basis of modern electrowetting was first described in detail by G. Lippmann in 1875 [9]. In 1993, Berge et al. introduced the concept of electrowetting on dielectric (EWOD), in which a thin insulating layer is used to separate the conductive liquid from the electrode, preventing electrolysis [10]. Subsequent to these discoveries, much research has focused on optimizing the insulating layer to improve the performance and utility of electrowetting systems [11,12,13].

In an EWOD system, three phase contact lines between the ambient air, surface liquid droplets, and the bottom dielectric layer initially stay within a force-balanced state to form a water/air contact angle *θ*_0_. When a voltage is applied to the dielectric layer, more and more charges accumulate on the surface of the dielectric layer, especially on the interface between the liquid droplets and the dielectric layer, leading to the contact angle decreasing from *θ*_0_ to *θ_V_* [14]. The dependence of the contact angle (*θ_V_*) on the applied voltage (*V*) is given by Lippmann’s equation [1,9,10]:

(1) $$\cos\theta_{V}=\cos\theta_{0}+\frac{\varepsilon_{0}\varepsilon_{r}}{2d\gamma }V^{2},$$

where *θ*_0_ is the initial contact angle, *γ* is the surface tension of the liquid (or interfacial tension between two fluids), *ε_0_* is the vacuum permittivity, *ε_r_* is the relative permittivity of the insulator, and *d* is the thickness of the insulator. The commonly referred to EW number, *η* = *ε*_0_*ε_r_V*^2^*/*2*dγ*, is a dimensionless number that indicates the change in contact angle under an applied voltage.

The dielectric layer is crucial to the EWOD system because it affects some key properties, including driving voltage, electrowetting degradation, and leakage current [15,16,17]. Insufficient dielectric strength leads to a breakdown of the dielectric layer before it reaches the working voltage [18], and a hydrophobic dielectric layer surface is required for larger variations of the contact angle. Amorphous fluoropolymers have often been applied as insulating and hydrophobic layers [4,15,19] or as hydrophobic top coatings combined with inorganic insulating layers beneath. The inorganic materials used for insulating coatings include SiO_2_, TiO_2_, Si_3_N_4_, Al_2_O_3_, etc. [20,21,22]. In the recent literature, there are studies providing investigations into novel ultrahigh-voltage insulating materials [23,24]. These have potential for use as the dielectric layer in future electrowetting studies, but this is beyond the scope of the present work. Cytop and Teflon AF are currently the two most popular candidates because of their low surface energy and good film-forming ability [13,25]. The high chemical stability of fluoropolymers in harsh conditions is also beneficial for related electrowetting applications. In addition, the solution processability of Cytop and Teflon AF results in the easy fabrication of films, and is compatible with a large-scale printing approach for film coatings [4].

**Figure 1:**
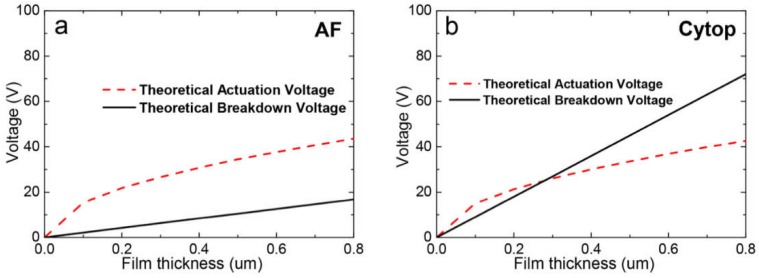
The calculated electrowetting actuation voltage (*E_w_* = 0.34) and the theoretical breakdown voltage, dependent on film thickness, of (**a**) Teflon AF coatings and (**b**) Cytop coatings.

Cytop possesses a much higher dielectric strength (90–110 V/μm) than Teflon AF (21 V/μm) [13]. It also shows a relatively high breakdown voltage and superior long-term electrowetting for dielectric performance [26]. Figure 1 shows the dependence of both electrowetting actuation voltage and theoretical breakdown voltage on film thickness. This was calculated using Lippmann’s equation (Equation (1)) with *E_w_* equal to 0.34, which provides a ~20° water/air contact angle variation on the fluoropolymer surface. The breakdown voltage of Teflon AF film is clearly much lower than the actuation voltage for the thickness range 0–0.8 μm. Conversely, the breakdown voltages of Cytop films are only lower than the actuation voltage before the film thickness reaches ~0.37 μm. Theoretically, once the films are thicker than ~0.37 μm, the breakdown voltages become increasingly higher than the actuation voltage.

The surface of Cytop is less hydrophobic than that of Teflon AF. Numerous –CF_3_ groups lower Teflon AF’s surface energy, producing a higher initial contact angle, larger contact angle variation under applied voltage, and better EW device performance than Cytop, which contains only –CF_2_ groups [27].

In this work, we integrated the advantages of the two fluoropolymer candidates into a composite layer, namely, an insulating Cytop bottom layer and a hydrophobic Teflon AF top coating. The electrowetting and leakage current of films with both single and composite materials were investigated. In contrast to the fabrication of parylene or inorganic materials, which require extra techniques that are more complex and more inefficient than wet coating (e.g., spin coating) [12], this stack maintained good dielectric strength, created a more hydrophobic surface, and was easy to fabricate, making it greatly superior to parylene and fluoropolymer-integrated films. The formation of this composite provides a simple and practical strategy for the creation of a workable and robust dielectric layer in electrowetting systems.

## 2. Materials and Methods

### 2.1. Materials

Teflon AF1600X (Chemous, Shanghai, China) was dissolved in fluorinate electronic liquid (FC-43; Minnesota Mining & Manufacturing Company, Saint Paul, MN, USA) with a concentration of 2–4 wt.% as a key raw material. Cytop 809A (Asahi Glass Co., Ltd., Kanagawa, Japan) was dissolved in CT-SOLV180 (Asahi Glass Co., Ltd.) with a concentration of 4–7 wt.% as another key raw material. As the substrate and bottom electrode, indium tin oxide (ITO) glass (electrical resistance: 100 Ω/□; Guangdong Jimmy Glass Technology Ltd., Foshan, China) was fully cleaned by a commercial cleaning line (KJD-7072ST, KEJINGDA Ultrasonic Equipment Co., Ltd., Shenzhen, China) prior to use.

### 2.2. Dielectric Layer Preparation

**Figure 2:**
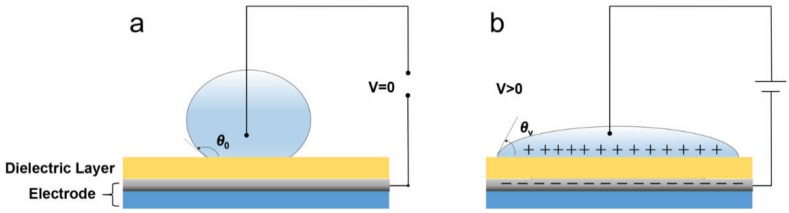
Schematic of the electrowetting on dielectric (EWOD) principle. (**a**) Initial condition of water droplet with no voltage applied. *θ*_0_ is the initial water/air contact angle. (**b**) Water droplet shape and water/air contact angle (*θ_V_*) after voltage was applied.

The structures of the samples are shown in Figure 2. A fluoropolymer solution was spin-coated on the surface of ITO glass using a spin coater (KW-5, Institute of Microelectronics Chinese Academy of Sciences, Beijing, China) at 1000–3000 rpm for 60 s and then dried on a hotplate at 85 °C for 3 min and in an oven (101-5B, SUBO Co., Ltd., Shaoxing, China) at 185 °C for 1 h. For the composite layers, all fabrication methods were the same as above.

### 2.3. Characterizations

The thickness of the fluoropolymer film was determined by a stylus profiler (Dektak XT, BRUKER, Shanghai Office, China). The water contact angle on the surface of the fluoropolymer film was measured by a contact angle meter (POWEREACH, Shanghai Zhongchen Digital Technology Apparatus Co., Ltd., Shanghai, China). Atomic force microscopy (AFM) images were taken by a MultiMode8 (Bruker, Guangzhou, China) with a monocrystalline cantilever of Bruker ScanAsyst at a force of 0.8 nN. Instantaneous current line was recorded by a picoammeter (Keythley 6487, Cleveland, OH, USA) with a platinum-coated needle using a 10-μL NaCl aqueous droplet (0.01 mol/L) on the film surface as the top electrode (see Figure 2). The capacitance value of the fluoropolymer film was measured with an impedance analyzer (WAYNE KERR 6500, Chichester, UK) using a needle-inserted 10-μL NaCl aqueous droplet to calculate the dielectric constant.

## 3. Results and Discussion

**Figure 3:**
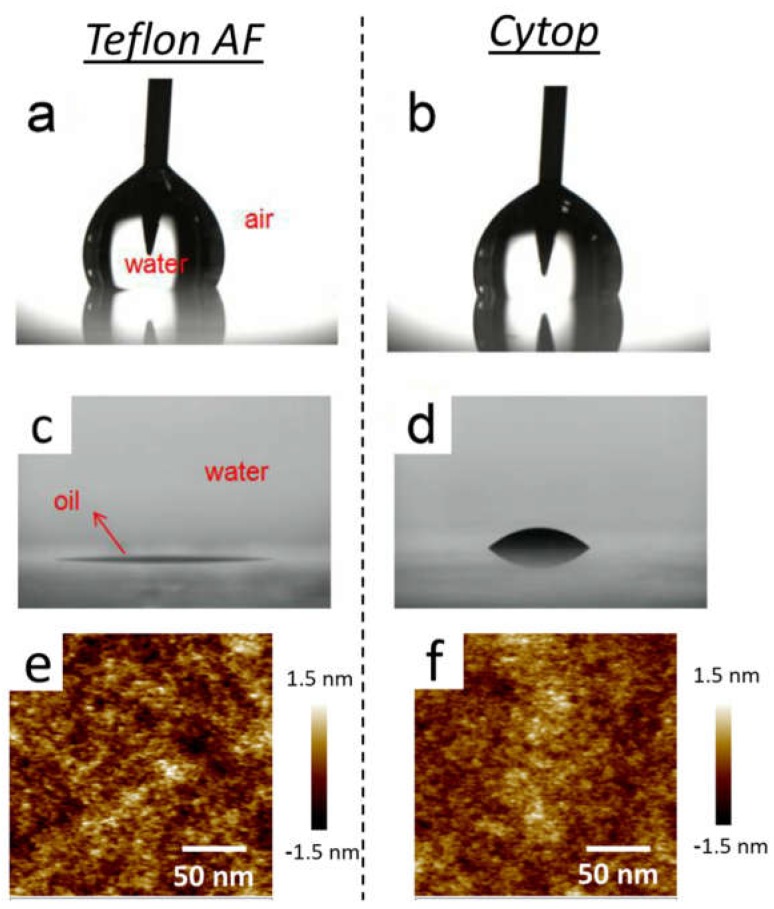
Water/air contact angle on the coating of (**a**) Teflon AF and (**b**) Cytop. Oil/water contact angle on the coating of (**c**) Teflon AF and (**d**) Cytop. AFM image of (**e**) Teflon AF surface and (**f**) Cytop surface.

To determine surface wettability, we first measured the contact angle on the surfaces of Teflon AF and Cytop films. Regarding the water/air contact angle (*θ*_water/air_), Teflon AF’s was ~120°, approximately 10° higher than Cytop’s. For the oil/water contact angle (*θ*_oil/water_), Teflon AF’s had an upper limit of 5° but Cytop’s reached ~40°. The contact angles are shown in Figure 3a–d. The surface topographies of the two films detected by AFM are shown in Figure 3e,f. According to the AFM images, the surfaces of the two coatings were both flat, and the average roughness of the Teflon AF and Cytop coatings were 0.33 nm and 0.27 nm, respectively. This indicated that the surface wettability difference could be attributable to the chemical properties of the coatings rather than their surface topographies.

It has been reported that Cytop is not hydrophobic enough for electrowetting display (EWD) [27] because the response speed of EWD is mainly determined by dielectric surface hydrophobicity. After the introduction of Teflon AF, the fast response time of EWD devices with ~10 ms was achieved [4,27]. In 2017, Han Zhang et al. provided a sacrificial strategy for electrowetting arrays to enhance the surface hydrophobicity of Cytop to almost the maximum extent, but this only contributed up to 40 ms to the response time [28]. Regardless of which EWD application is put into practice, all basic electrowetting systems require the highest possible surface hydrophobicity to achieve larger variations of the contact angle and satisfactory reversibility [29].

**Figure 4:**
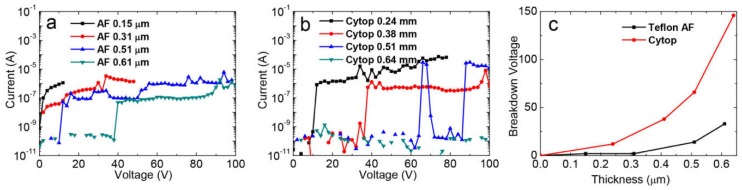
The dependence of leakage current on the voltage of (**a**) Teflon AF coatings, and (**b**) Cytop 809A coatings, of various thicknesses, as well as (**c**) the breakdown voltage of Teflon AF and Cytop 809A, dependent on film thickness.

For dielectric properties, we investigated the leakage currents of Teflon AF and Cytop films with different thicknesses. As seen in Figure 4a,b, the fluoropolymer coatings showed good insulating properties with a leakage current of less than 10^−9^ A under low applied voltage. Despite this, once the voltage continued to increase, a sharp increase in current occurred, indicating failure or breakdown [11]. At this stage, this voltage is viewed as the breakdown voltage. Figure 4c shows the relationship between the breakdown voltage and the film thicknesses of Teflon AF and Cytop. Here, the dielectric breakdown voltage of the Teflon AF film with a thickness of 0.51 μm was 12 V, which was equivalent to the electric field (*E*) at the breakdown point of 23 V/μm. These values were obviously higher than for a thinner Teflon AF film with a thickness of 0.31 μm (with breakdown voltage of 2 V and *E* of 6 V/μm). The breakdown voltage of the Teflon AF coating with a thickness of 0.61 μm even reached 40 V (an *E* of 66 V/μm). This matches findings from Hayes’ report [15]. Compared with the Teflon AF coatings, the Cytop coatings exhibited much better dielectric properties. For a 0.24-μm-thick Cytop coating, the breakdown voltage was 12 V (an *E* of 50 V/μm), and for a 0.64 μm coating, the breakdown voltage increased significantly to a value of 148 V (an *E* of 231 V/μm).

**Figure 5:**
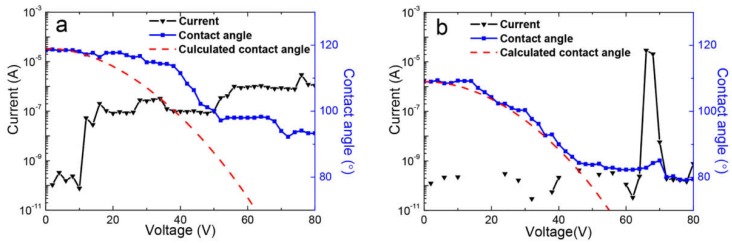
Leakage current and electrowetting measurement of (**a**) Teflon AF coating with a thickness of 0.51 μm and (**b**) Cytop 809A coating with a thickness of 0.51 μm. Measured current in black (left axis) and contact angle data in blue (right axis) with Equation (1) modeled (red dashed line).

Hayes reported [15] that the loss factor (tan ð) was low for a 0.5-μm-thick Teflon AF coating, and that the dependence of the contact angle curve on voltage agreed with Lippmann’s equation, but the electrowetting curve of the coating quantitatively deviated from the theoretical value. We observed a similar phenomenon for 0.51-μm-thick Teflon AF (Figure 5a (right vertical axis)) as well as for the leakage current curve (Figure 5a (left vertical axis)). Here, the turning point of the electrowetting curve was strongly affected by the current value. The first increasing range of leakage current, from less than 10^−9^ A to approximately 10^−7^ A, with an applied voltage of 12 V (an *E* of 23 V/μm), promoted electrowetting curve deviation from the theoretical value. By contrast, the second increasing range of leakage current at 52 V (an *E* of 102 V/μm) caused the electrowetting curve slope to decrease and then plateau. The theoretical electrowetting curves were calculated by Lippmann’s equation (Equation (1)). For comparison, the electrowetting behavior of the Cytop coating with a thickness of 0.51 μm was also measured. When the applied voltage was less than 44 V (an *E* of 86 V/μm), the measured and theoretical contact angle values fitted well together. When the applied voltage was 46 V (an *E* of 90 V/μm) and was slightly increased to 62 V (an *E* of 122 V/μm), the contact angle curve reached a plateau at ~86°, and breakdown occurred following this. The slight deviation of the contact angle curve from the theoretical curve under 12 V (an *E* of 24 V/μm), 28 V (an *E* of 55 V/μm), and 38 V (an *E* of 75 V/μm) may be attributable to charge injecting or trapping. Compared with the 0.51 μm Teflon AF coating, the Cytop coating with the same thickness showed a better electrowetting ability (Figure 5b).

**Figure 6:**
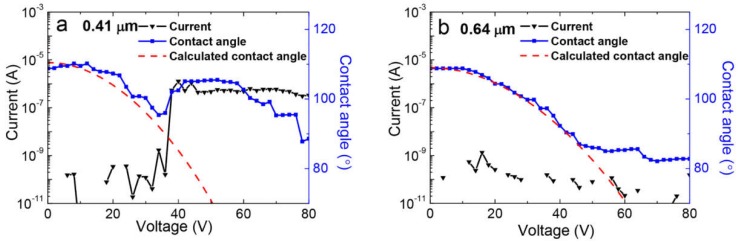
Leakage current and electrowetting measurement of Cytop coatings with thicknesses of (**a**) 0.41 μm and (**b**) 0.64 μm. Measured current in black (left axis) and contact angle data in blue (right axis) with Equation (1) modeled (red dashed line).

The electrowetting behavior of Cytop coatings with different thicknesses was also studied (Figure 6a,b). For the 0.41 μm Cytop coating, contact angle recession occurred at the film breakdown point under the applied voltage of 38 V (an *E* of 93 V/μm) along with a rising leakage current. At this point, the breakdown voltage was lower than the saturation voltage of the film. However, when the applied voltage was less than 46 V (with a saturation voltage of 48 V, a breakdown voltage of 148 V, and an *E* of 231 V/μm), the electrowetting response curve of the 0.64 μm Cytop coating agreed with the calculated curve, and the contact angle remained stable at ~88°. In addition, the leakage current stayed at a low value before the saturation point was reached.

**Figure 7:**
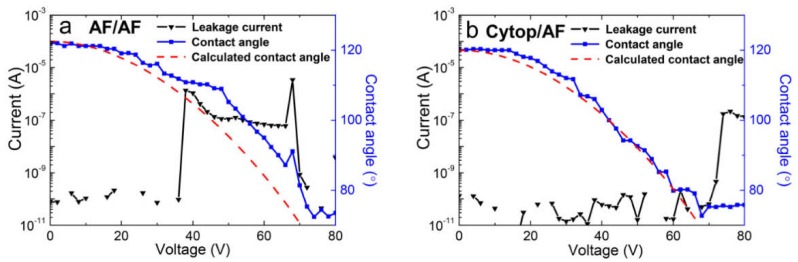
Leakage current and electrowetting measurement of bilayer fluoropolymer coatings with (**a**) 0.52 μm Teflon AF as the bottom layer and 0.09 μm Teflon AF as the top layer; and (**b**) 0.5 μm Cytop as the bottom layer and 0.06 μm Teflon AF as the top layer. Measured current in black (left axis) and contact angle data in blue (right axis) with Equation (1) modeled (red dashed line).

Based on the above results, we concluded that coatings with a thickness of ~0.5 μm are sufficient for electrowetting in a water/air environment. Following this, a thin top layer of Teflon AF (0.06 μm) was placed onto the 0.5-μm-thick Cytop layer. Figure 7b shows that the breakdown voltage was 72 V (an *E* of 129 V/μm), close to that of the 0.51 μm Cytop coating with a breakdown voltage of 66 V (an *E* of 129 V/μm). The initial contact angle on the stack surface increased to ~120°, which could be due to the more hydrophobic surface of the Teflon AF top coating. In addition, the contact angle variation (Δ*θ*) of the Cytop/Teflon AF composite film rose to ~45°, which was much greater than that of the single-layer Cytop coating (~28°). This may also be the result of the high charge storage capability of the bottom Cytop coating. As reported previously, the charge storage capacity of Cytop electrets is higher than that of Teflon AF [25]. We also carried out electrowetting and leakage current tests on the bilayer Teflon AF coating, as shown in Figure 7a. Noticeably, the withstand voltage did not show an obvious difference from that of the single-layer Teflon AF coatings, and the contact angle showed a deviation starting only from the film breakdown voltage.

**Figure 8:**
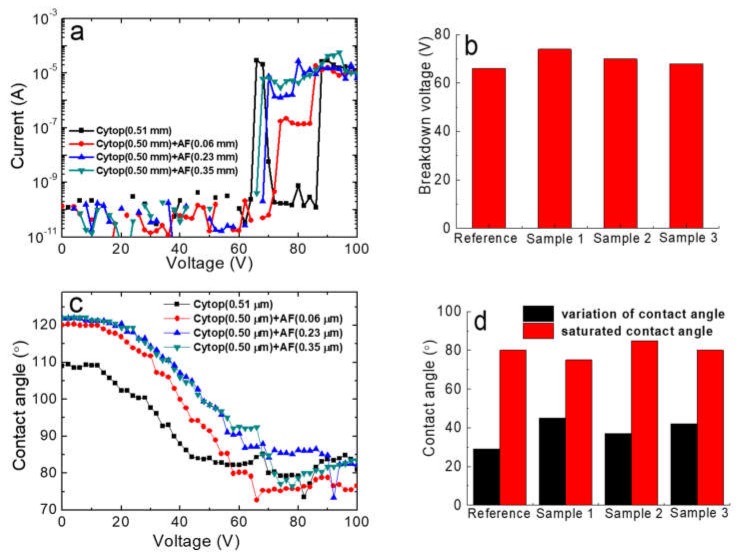
(**a**) Leakage current measurement, (**b**) breakdown voltage, (**c**) electrowetting measurement, and (**d**) contact angle variation and saturated contact angles of the single-layer Cytop coating reference samples. Sample 1: 0.5 μm Cytop bottom coating and 0.06 μm Teflon AF top coating; Sample 2: 0.5 μm Cytop bottom coating and 0.23 μm Teflon AF top coating; Sample 3: 0.5 μm Cytop bottom coating and 0.35 μm Teflon AF top coating.

To further investigate the effect of the Teflon AF top layer, we constructed three composite films containing a Cytop bottom layer (0.50 μm) and a Teflon AF top layer with different thicknesses. The dependencies of current and contact angle on voltage are shown in Figure 8. The breakdown voltage of the composite films was within the range 68–72 V, which was not different from the 66 V breakdown voltage of the single-layer Cytop coating. The strong hydrophobicity of the top Teflon AF coatings greatly contributed to the initial contact angles of ~120°, which were higher than those of the single-layer Cytop coating (~110°). The saturated contact angle of the composite films was 75–85°, which was similar to that of the single-layer Cytop coating. Compared with a contact angle variation of only ~28° for the single Cytop layer, those of the composite coatings were as large as 36–46°. According to Figure 8, the increased thickness of the top Teflon AF coating did not increase its electrowetting ability.

**Figure 9:**
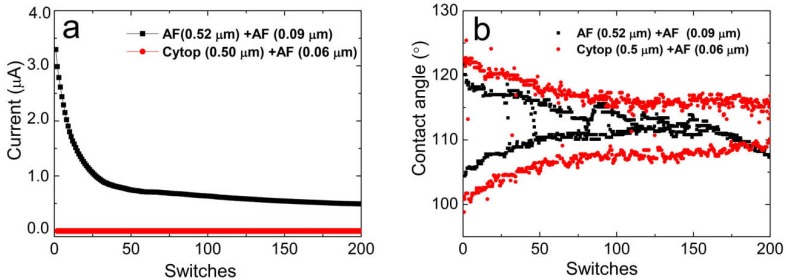
Leakage current and electrowetting reduction of bilayer fluoropolymer coatings with (**a**) 0.52 μm Teflon AF as the bottom layer and 0.09 μm Teflon AF as the top layer, and (**b**) 0.5 μm Cytop as the bottom layer and 0.06 μm AF as the top layer.

Finally, we performed comparative durability tests with 200 switches facing two-layer Cytop/Teflon AF and Teflon AF/Teflon AF coatings. As shown in Figure 9, 40 V was applied to the films for 200 switches, with an on/off time of 3 s. For the entirety of the test, the leakage current of the Cytop/Teflon AF coating was lower than 10^−9^ A, without breakdown failures. The equivalent conductivity of the Cytop/Teflon AF coating was lower than 1.71 × 10^−12^ S/m. Note that this conductivity value is limited by the accuracy of the equipment and the current rearmament setup. The area of the water droplet/fluoropolymer interface for the conductivity calculation was achieved by geometric methods based on a spherical cap with the given value of the droplet volume and contact angle. The leakage current of the two-layer AF coating was within the range of 1 x 10^−7^ to 4 × 10^−6^ A. The conductivities were within the range of 2.85 × 10^−10^ to 1.14 × 10^−8^ S/m. Additionally, ITO corrosion within the testing area was observed by microscope.

These tests verified the breakdown failure of two-layer AF coatings. There was also a leakage current decrease for the AF coating during testing. This may be due to the gradual charge injection saturation of the film and the increasing failure of ITO to further restrain more charges being injected or transferred through the film.

Figure 9b shows that the initial contact angles (*θ*_0_) of the two films at 0 V were ~122°, but the initial contact angles (*θ_v_*) of the Cytop/Teflon AF coating and of the Teflon AF/Teflon AF coating at 40 V were ~101° and ~105°, respectively. Particularly, the initial *θ_v_* showed a smaller wetted angle to both the Cytop/Teflon AF and Teflon AF/Teflon AF cases. Mibus et al. observed the same phenomenon and ascribed it to variability in contact angle saturation [22]. The *θ*_0_ of the Cytop/Teflon AF coating gradually decreased from 122° to 117° during the initial ~80 switches and then plateaued at ~117°. In contrast, the *θ*_0_ of the Teflon AF/Teflon AF coating dropped sharply from ~122° to 118° in the first five switches and gradually decreased to ~110°. On the other hand, there was a fluctuation around the 80-switch mark for the Cytop/Teflon AF coating. This rising *θ*_v_ may be related to the transitory release of injected charges. After ~120 switches, there was still an ~8° contact angle variation remaining for the on/off state (Δ*V*_on/off_), while the Δ*V*_on/off_ of Teflon AF/Teflon AF coatings was nearly 0°. The degradation of the contact angle during the durability tests can be explained by charge injection theory [11,26]. The situation was made worse for the Teflon AF/Teflon AF coating because of film breakdown failure. As testing continued, the breakdown failures increased further, finally resulting in film disfunction.

## 4. Conclusions

In this project, we combined the advantages of two fluoropolymers and investigated the electrowetting of Cytop/Teflon AF composite nanolayers. Cytop coatings with a thickness of 0.5 μm and a breakdown voltage of 66 V (an *E* of 132 V/μm) were found to be satisfactory due to their dielectric strength. A thin 0.06 μm Teflon AF top coating was used to enhance the surface hydrophobic properties and increase the *θ*_water/air_ to ~120°. Thicker Teflon AF coatings were tried, but they did not noticeably improve the Cytop/Teflon AF stack. The contact angle tunable ranges of 35–45°, produced by the electrowetting of Cytop/Teflon AF composite films, were larger than those of single-layer Cytop coatings of only ~25°. Finally, we compared the Cytop/Teflon AF composite coating and the Teflon AF/Teflon AF two-nanolayer coating and found that the withstand voltage and endurance of the composite film were superior to those of the Teflon AF two-layer coating.
